# Community Pharmacists’ Opinions towards Poor Prescription Writing in Jazan, Saudi Arabia

**DOI:** 10.3390/healthcare9081077

**Published:** 2021-08-21

**Authors:** Saad Saeed Alqahtani

**Affiliations:** 1 Department of Clinical Pharmacy, College of Pharmacy, Jazan University, Jazan 45142, Saudi Arabia; ssalqahtani@jazanu.edu.sa; 2 Pharmacy Practice Research Unit, College of Pharmacy, Jazan University, Jazan 45142, Saudi Arabia

**Keywords:** e-prescription, prescription writing, Jazan, Saudi Arabia, prescription errors

## Abstract

Avoidance of medication errors is imperative for the safe use of medications, and community pharmacists are uniquely placed to identify and resolve the errors that may arise due to poorly handwritten prescriptions. **Purpose:** To explore the opinion and attitudes of community pharmacists towards poor prescription writing and their suggestions to overcome this concern. **Methods:** A cross-sectional, self-administered survey was conducted among the community pharmacists in the Jazan region, Saudi Arabia. Descriptive analysis and chi-square test were used at 5% *p*-value (*p* > 0.05) as the significance level. **Results**: The response rate for the survey was 78.66%, and 140 community pharmacists agreed to participate. Among the study subjects, the majority (73.57%) had a bachelor’s degree. Nearly three-fourths (3/4) of the pharmacists (72.29%) chose to send the patient back to the prescriber when they found difficulty in interpreting the information from an illegible prescription. As many as 80.71% of the pharmacists believed that poorly handwritten prescriptions were the cause of actual errors when dispensing medications. The most commonly encountered problem due to poorly handwritten prescriptions was the commercial name of medicine, which was reported by around two-thirds (67.86%) of the pharmacists. The use of e-prescription was suggested by 72.86% of the pharmacists as a probable solution to encounter this problem. **Conclusion**: Our findings highlight the belief and attitudes of community pharmacists in the region and their opinions to solve this impending problem of poor prescription writing. Continuous professional development courses can be adopted to tackle the problem. Additionally, health authorities can work on incorporating and facilitating the use of e-prescription in the community sector, which can be a boon to physicians, pharmacists, and patients. Proper and extensive training is however needed before the implementation of e-prescribing.

## 1. Introduction

The community pharmacist is usually the first point of contact for people due to their easy accessibility. They dispense medications as stated in the prescription and are licensed to prescribe over-the-counter drugs [1]. Nowadays, community pharmacists also contribute professionally through a wide range of activities that concern patient care from the optimization of drug therapy to promote health awareness and to educate people on the prevention of diseases. All this is not to mention their essential role in providing rational drug information and to counsel patients about drug safety and cost-effectiveness [2]. One of the primary tasks of a pharmacist is to verify the legality, safety, and appropriateness of the prescription and to ensure accurate dispensing of the medication before deciding to hand it over to the patients with directions of use and counselling [1].

The WHO’s Guide to Good Prescribing states “A prescription is an instruction from a prescriber to a dispenser” [3]. The word *prescription* stems from the Latin language, wherein *pre*- translates as “before” and *scribe-* translated for writing [4]. A written prescription is the physician’s/prescriber’s order to the dispenser, usually for a pharmacist to prepare and/or dispense the specified medication to that patient [5]. Almost all interactions between a doctor and a patient end with prescription writing [6], therefore making it imperative that the prescriber should always ensure the legibility and unambiguity of the written order, including the date and sign. This facilitates clear communication between health care professionals. Moreover, an ideal prescription should also present ample information to allow the dispenser (in most cases the pharmacist) to identify any errors before dispensing [5].

A medication error can be defined as an unwanted event that may lead to inappropriate use of medications and potentially be harmful to the patients [7,8]. According to world estimates, the cost of medication errors is around 42 billion US dollars [9]. These medication errors are often preventable if identified at the right time [7]. Although medication errors occur at different stages such as writing, transcribing, and administration, illegible handwriting appears to be the predominant cause of these errors [10]. 

Bobb et al. (2004), Delgado Silveira et al. (2007), and Aljadhey et al. (2013) reported that the point of prescribing medication is usually linked to a high incidence of medication errors, which in turn is the leading cause of adverse drug events [11,12,13]. More importantly, the illegibility of prescriptions leads to a greater chance of errors, whether or not the written order is complete and accurate. Illegible prescription is one of the factors that can increase the risk of medication errors regardless of the accuracy and completeness of the prescription [14]. Analysis of self-reporting was done by Knudsen et al. (2007) in community pharmacies, and they found a positive correlation between dispensing errors and illegible handwriting [15].

A study by Hartel et al. (2011) evaluated and noted significant variation in the legibility and quality of the handwriting of prescribers and concluded that there are differences in the ability of pharmacists to read these written orders [16]. Additionally, Brits et al. (2017) demonstrated in their study that community pharmacists were not better than nurses or physicians in reading the prescription, and they attributed this to the lack of direct work associated with the doctors [17]. In a similar Saudi study done by Albarrak et al. (2014), pharmacists with less experience found difficulty in reading 21.6% of prescriptions as opposed to experienced pharmacists (2%) [18]. Winslow et al. (1997) reported that 20% of prescriptions had poor handwriting and could not be understood [19]. In a study done in Saudi Arabia by Irshaid et al. (2005), around 64% of medication orders were illegible [20]. Calligaris et al. (2009), after evaluating the prescriptions in an Italian hospital confirmed that 24% of them were illegible [10].

In view of the above evidence, critically addressing the illegibility problem in medication orders is the need of the hour. The current study aimed at exploring the attitude of the community pharmacists towards poor prescriptions in the Jazan region of Saudi Arabia. We also investigated the prescription-related problems due to poor handwriting and suggestions. Lastly, we aimed to garner suggestions from the community pharmacists about improving the quality of handwritten prescriptions.

## 2. Methodology

### 2.1. Ethics Approval

The study was approved by the Institutional Research Review and Ethics Committee (IRREC) of the Faculty of Pharmacy, Jazan University, KSA. The study protocol was in accordance with principles and guidelines laid out in the Declaration of Helsinki and the International Council on Harmonization Good Clinical Practice. All pharmacists were asked to complete a written consent form prior to the start of the survey.

### 2.2. Study Design and Area

This was a cross-sectional, structured, self-administered survey of the belief, attitudes, and suggestions of pharmacists about poor prescription writing in the Jazan province of Saudi Arabia. Jazan is a province located in the Southwestern part of the Kingdom with a total population of 1,535,167 (2016), and the city of Jazan serves as its administrative headquarter [21].

### 2.3. Study Population and Sample Size

The questionnaire (Supplementary Materials) was distributed among licensed pharmacists working in community pharmacies in different areas of Jazan province, and the data were gathered through an anonymous, self-administered questionnaire. The selection of licensed pharmacists working in both independent and chain pharmacies was done randomly. After obtaining their consent, the questionnaire was delivered to them and was collected the next day by a research assistant. A total of 140 community pharmacists agreed to enroll in the study from the Jazan province. 

### 2.4. Data Collection Tool

The questionnaire was face-validated by a five-member expert panel prior to the study. The panel comprised one English language expert, one psychologist, two practicing community pharmacists, and one academic pharmacist. The two practicing community pharmacists were excluded from the study. The questionnaire was in the English language and included the demographic information of the respondents along with their education level and ownership details. The community pharmacists were further asked about the number of prescriptions filled by them per day and the number of poorly written prescriptions received per day. The respondents were also asked for their opinion about handwritten prescriptions, their related errors, and the action to be taken.

The second part of the questionnaire was designed to identify the most common prescription-related problem that arises due to poor handwritten prescriptions. The last part of the questionnaire explored the measures that were suggested by the pharmacists to minimize the errors due to illegible prescriptions.

### 2.5. Statistical Analysis

The items in the questionnaire were first coded and then entered into Microsoft Excel. The data were then analyzed on STATA (Version 15.0 software, Stata Corp LP, College Station, TX, USA), and descriptive analysis (frequencies and percentages) was performed for all variables included in the study. Chi-square test was employed for categorical data and significance was considered if the *p*-value was less than 0.05 (5% *p*-value)

## 3. Results

### 3.1. Demographic Data

Out of 178 community pharmacists approached for the study, 140 (78.66 %) consented for enrollment in this study. The mean age of participants was 31.9 years. More than half of the respondents were found to be less than 30 years (62.75%) and had less than 10 years of experience (62.06%), with the mean experience as 8.5 years. Regarding the level of education, around three-fourths of pharmacists (73.57%) held a Bachelor’s of Pharmacy degree. Nearly all of the respondents (97.13%) were working as employees in the private sector. The detailed demographic data are presented in Table 1.

### 3.2. Prescriptions

A total number of 2762 prescriptions (mean: 19.58 prescriptions per pharmacist) were dispensed by all the responding pharmacists. The number of prescriptions dispensed on a daily basis were stratified into two groups: <50 and ≥50. The majority of community pharmacists (92.41%) were found to fill less than 50 prescriptions on daily basis. As many as 93.79% of community pharmacists reported that they received around 30 poor handwritten prescriptions per day (Table 1).

### 3.3. Response of the Pharmacists

When community pharmacists were asked about their response upon receiving an illegible prescription, nearly three-fourths (72.29%) of the pharmacists preferred to return the patient back to the physician when they could not interpret the information from the prescription. Around 93% of the pharmacists responded that they would never tell the patient that the medication was not available when they were not able to read the name of the medication. We only found a significant association between the experience of the pharmacist and the variable “I cannot read the prescription” (*p* = 0.008) (Table 2). No association was found between the educational degree of the pharmacist and their response to poor prescriptions.

### 3.4. Belief of the Pharmacists

**Figure 1:**
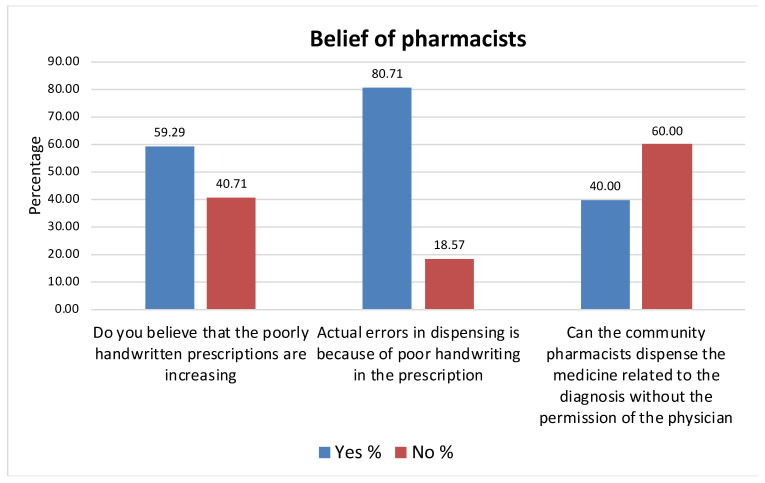
Beliefs of pharmacists of poorly handwritten prescriptions.

About 60% of the pharmacists believed that poorly written prescriptions are increasing. In addition, the majority of pharmacists (80.71%) thought that actual errors when dispensing medications were due to the poor handwriting in the prescription. Sixty percent of the community pharmacists had the belief that the community pharmacist should not dispense the medication based on diagnosis without consulting the physician (Figure 1).

### 3.5. Prescription-Related Problems Due to Poor Handwriting

**Figure 2:**
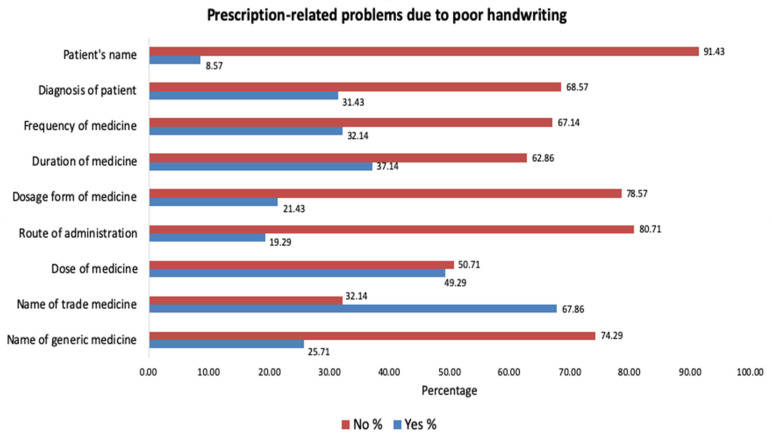
Problems encountered due to poor handwritten prescriptions.

Around two-thirds (67.86%) of the respondents reported that the name of the trade medicine was the common prescription problem encountered due to poor handwriting followed by dose of the medication (49.29%). Of the nine items that were asked, the patient’s name was the least reported (8.57%) to be a problem due to illegible handwriting (Figure 2).

### 3.6. Actions Suggested by the Community Pharmacists

Around three-fourths (72.86%) of the respondents suggested the use of e-prescription, and this only had a significant association with the educational degree of the pharmacist (*p*-value = 0.002) (Table 3). Most of the community pharmacists (90.27%) did not think that decimal numbers should be avoided when writing the dose of medications. Additionally, more than three-fourths (78.57%) did not suggest writing in capital letters, followed by two-thirds (66.43%) who did suggest the need for introducing a structured prescription form. There was no association between any of the actions suggested by the pharmacists and their experience. However, there was a significant association (*p* = 0.002) between the educational degree of the respondents and the suggestion to use e-prescriptions (Table 4).

## 4. Discussion

The present study is the first of its type to evaluate the attitude and belief of the pharmacists about poorly handwritten prescriptions in the Jazan region of Saudi Arabia. Poorly written prescriptions can cause errors that can lead to some serious consequences for the patient. An appropriately written prescription is a result of not only the effort by a prescriber to minimize errors but also to strive to achieve better prescribing [22]. Lopes et al. had reported that most medications errors reported in community pharmacies are due to poorly handwritten prescriptions [23]. In our study, nearly 80% of the pharmacists preferred to return the prescription to the physician for review. This seems to be the right decision by the pharmacists, as the prescribing physician can re-write the prescription or clarify the concern related to the prescription. This will prevent any unwanted dispensing error and will also be a reminder to the physician to be more legible. Moreover, the experience of the community pharmacist had a statistically significant association (*p* = 0.008) with the response “I cannot read the prescription”. This seems to be logical as community pharmacists who are experienced will not respond that they cannot read the prescription, as this response would affect the confidence and trust between the patient and the pharmacist.

Nearly 80% of our respondents believed that the actual errors in dispensing are because of poor handwriting. A recent study done by Al-Arifi in Central Saudi Arabia reported that around 55% of the community pharmacists had a perception that dispensing errors are most common, and poor handwriting was identified as one of the major causes [24]. An earlier study by Knudsen et al. in Denmark also identified handwritten prescriptions as one of the four causes for the increase in dispensing errors [25].

In our study, nearly 68% reported that trade medicine was a major problem in poorly written prescriptions. This is consistent with many studies where the use of the trade name of the medicine was one of the main contributors to the prescription errors [10,24,25]. Moreover, nearly half (49.29%) of the respondents opined that the dose of the medication was also a concern in illegible prescriptions. This is much higher than the observations of Knudsen et al., who reported errors in dosages of 37.4%.

Community pharmacists in our study were also asked for their suggestions to improve the quality of prescriptions. The majority of them (72.86 %) suggested the use of e- prescriptions as a solution to the problems arising due to poor handwriting. There was a statistically significant association (*p* = 0.002) between the educational degree of the pharmacist and their suggestion to use e-prescription (Table 3). No significant association was found between the years of experience as a pharmacist and the suggestion to use e-prescription. The use of electronic prescribing can be a viable alternative that could reduce the incidence of prescribing errors. Various studies have shown that e-prescription smoothens the dispensing process compared to handwritten prescriptions due to their better completeness, clarity, and legibility [25,26,27]. However, the implementation of e-prescriptions seems to be a problem in the community pharmacy settings in Saudi Arabia. Although major hospitals and specialist centers in Saudi Arabia practice e-prescribing [28], there is still a need for implementation in public health centers and the private health sector. Along with the challenges pertaining to implementation, e-prescribing is not void of its own share of limitations. 

The present study highlights the responses of the community pharmacists and prescription-related problems due to poor handwriting in the Jazan region of Saudi Arabia. The data from this study can be used as baseline data to elicit further research into the barriers to e-prescribing in private physician practice and integration with the community pharmacies. However, our study had its own share of limitations. The results from our study cannot be generalized, as we used a convenience random sampling technique and the data pertain to a single province in Saudi Arabia. It would be of great benefit to conduct a similar nationwide survey as the results would then be generalizable and will aid the healthcare authorities in making impactful decisions. Additionally, only descriptive analysis was performed due to the small sample size. The study could not investigate the pharmacist-dependent factors such as work stress, lack of time, and workload, which may have affected the response of the pharmacist upon receiving an illegible prescription.

## 5. Conclusions

Our findings concluded the belief and attitudes of the community pharmacists in the Jazan region of Saudi Arabia and their opinions to solve this impending problem of poor prescription writing. Electronic medical records, structured prescription forms, and educational training are some of the reasonable solutions for the current problem; however, this research intends to seek the attention of the health care authorities about the issues faced by community pharmacists due to poor prescription writing. Healthcare authorities should take the initiative to provide training workshops on proper prescription writing, as this would not only benefit physicians and pharmacists but also help safeguard patient safety. Future research can be targeted at recognizing the barriers in implementing e-prescribing as well as the use of printed prescriptions as it can highlight the roadblocks in the path of implementing a safe prescribing and dispensing environment.

## Figures and Tables

**Table 1:** Baseline data of the study subjects.

Variable	*n* = 140	%
**Age**		
>30 years	54	38.57
≤30 years	86	61.43
**Years of experience**		
<10 years	90	64.29
≥10 years	50	35.71
**Education Level**		
B. Pharm	103	73.57
Pharm. D	37	26.43
**Pharmacy Ownership**		
Employee	136	97.13
Owner	4	2.85
**Average prescriptions filled per day**		
<50	130	92.86
≥50	10	7.14
**Average number of poor handwritten prescriptions received per day**		
<30	132	94.29
≥30	8	5.71

**Table 2:** Comparison between the experience of the pharmacists and the response of the pharmacist.

Response of the Pharmacist	<10 Years		≥10 Years		*p* Value
	Yes	No	Yes	No	
Tell the patient this medication is not available.	4	86	7	43	0.092
Return the patient back to the physician.	69	21	42	8	0.419
I cannot read the prescription.	33	57	7	43	0.008 *

* *p* < 0.05.

**Table 3:** Actions suggested by pharmacists to overcome problems due to poor handwriting in prescriptions.

Suggestions by Pharmacists	Yes	No	Yes %	No %
Write in capital letters	30	110	21.43	78.57
Avoid abbreviations	64	76	45.71	54.29
Avoid the trade name of the medicine	57	83	40.71	59.29
Avoid the decimal number	13	127	9.29	90.71
Use e-prescription	102	37	72.86	26.43
Introducing a structured prescription form	47	93	33.57	66.43

**Table 4:** Comparison between the educational degree of the pharmacists and their suggestions to improve the prescriptions.

Suggestions by Pharmacists	B.Pharm		Pharm D		*p*-Value
	Yes	No	Yes	No	
Write in capital letters	21	83	9	27	0.711
Avoid abbreviations	48	56	16	20	1.0
Avoid trade name of the medicine	41	63	16	20	0.740
Avoid the decimal number	10	94	3	33	1.0
Use e-prescription	84	20	19	17	0.002 *
Introducing a structured prescription form	58	46	23	13	0.513

* *p* < 0.05.

## Data Availability

Data sharing not applicable.

## Supplementary Materials

The following are available online at https://www.mdpi.com/article/10.3390/healthcare9081077/s1, Community Pharmacists’ opinions towards poor prescription writing in Jazan, Saudi Arabia.
