# Supplementary material for: Community Pharmacists’ Opinions towards Poor Prescription Writing in Jazan, Saudi Arabia
Source: Healthcare (Basel). 2021 Aug 21;9(8):1077. doi: 10.3390/healthcare9081077 (PMC8391709; doi:10.3390/healthcare9081077)
Supplement: Supplementary file 1 [file healthcare-09-01077-s001.zip › healthcare-1286418-supplementary.pdf]

# Community Pharmacists' opinions towards poor prescription writing in Jazan, Saudi Arabia

Greetings! You are invited to participate in a study being conducted by Dr. Saad Alqahtani (Assistant Professor, Department of Clinical Pharmacy, College of Pharmacy, Jazan University).

The information collected in this survey will be used as part of a research study. There will not be any personally identifiable information collected in this survey, and all responses will be kept in the strictest confidence. Prior to analyzing the data, the order of respondents will be randomized to ensure your responses remain anonymous.

Your participation in this survey is voluntary. You may stop your participation in this survey at any time if you want.

The purpose of this study is to explore the opinions of the community pharmacists towards poor prescriptions in the Jazan region of Saudi Arabia.

If you have any questions or concerns regarding the study, please do not hesitate to contact me at ssalqahtani@jazanu.edu.sa

**PLEASE ANSWER THE QUESTIONS BELOW BEFORE PROCEEDING TO THE QUESTIONNAIRE**

***Please indicate by checking the box below that that you have read and agree to the above information.***

☐ I have read and understand the above information and AGREE to participate in this research study.

☐ I have read and understand the above information and DO NOT AGREE to participate in this research study.

# QUESTIONNAIRE

| DEMOGRAPHICS |
|---|
| 1) **Age in years** ☐ |
| 2) **Years of experience** ☐ |
| 3) **Educational Level** ☐ Pharm.D ☐ B.Pharm |
| 4) **Pharmacy Ownership** ☐ Employee ☐ Owner |
| 5) **Average number of prescriptions filled per day** ☐ |
| 6) **Average number of poor handwritten prescriptions received per day** ☐ |

| **Response on receiving a poor handwritten prescription** | | |
|---|---|---|
| | **YES** | **NO** |
| **7)** Tell the patient that the medication is not available. | ① | ⓪ |
| **8)** Return the patient back to the physician. | ① | ⓪ |
| **9)** Tell the patient, “I cannot read the prescription”. | ① | ⓪ |
| **Belief of the pharmacists regarding poor handwritten prescriptions** | | |
| **10)** Do you believe that the poorly handwritten prescriptions are increasing? | ① | ⓪ |
| **11)** Actual errors in dispensing is because of poor handwriting in the prescription. | ① | ⓪ |
| **12)** Can the community pharmacists dispense the medicine related to the diagnosis without the permission of the physician? | ① | ⓪ |

| **In your opinion, which of the following are the common prescription related problems due to poor handwriting?** | | |
|---|---|---|
| | **YES** | **NO** |
| **13)** Name of generic medicine | ① | ⓪ |
| **14)** Name of trade medicine | ① | ⓪ |
| **15)** Dose of medicine | ① | ⓪ |
| **16)** Route of administration | ① | ⓪ |
| **17)** Dosage form of medicine | ① | ⓪ |
| **18)** Duration of medicine | ① | ⓪ |
| **19)** Frequency of medicine | ① | ⓪ |
| **20)** Diagnosis of patient | ① | ⓪ |
| **21)** Patient's name | ① | ⓪ |
| **Please indicate your suggestions to overcome the problems due to poor handwriting in prescriptions.** | | |
| **22)** Write in capital letters | ① | ⓪ |
| **23)** Avoid abbreviations | ① | ⓪ |
| **24)** Avoid the trade name of the medicine | ① | ⓪ |
| **25)** Avoid the decimal number | ① | ⓪ |
| **26)** Use e-prescription | ① | ⓪ |
| **27)** Introducing a structured prescription form | ① | ⓪ |

☺ Thank you for completing this Survey ☺
